# NUMBER OF CHILDREN AND RISK OF ALZHEIMER’S DISEASE AND DEMENTIA: EVIDENCE FROM THE UK BIOBANK

**DOI:** 10.1093/geroni/igac059.2799

**Published:** 2022-12-20

**Authors:** Yan Zhang, Jason Fletcher

**Affiliations:** University of Wisconsin-Madison, Madison, Wisconsin, United States; University of Wisconsin-Madison, Madison, Wisconsin, United States

## Abstract

This study uses a large-scale dataset of half a million respondents aged 39–73 from the UK Biobank to examine how parity (i.e., number of children) may influence parents’ risk of Alzheimer’s Disease and dementia (AD/D). We use respondents’ (i.e., children’s) reports of their parents’ dementia status as the outcome variable. 38,040 respondents in the sample reported that their mother had AD/D, and 20,304 respondents reported their fathers had AD/D. Fixed effects logistic regression models suggest that compared to parents who had one child, high parity (> 4 kids) is associated with a lower risk of AD/D for both mothers and fathers. Moreover, as the parity increases, the protective effects become larger. This study advances prior dementia literature with two contributions. Methodologically, largely representative AD/D cases can increase the power of analysis. Also, children’s reported AD/D cases are very likely through the observation of parents’ entire life span, which can reduce misclassification of AD/D status and measurement errors from selective samples or self-reported cases. Empirically, this study provides important evidence suggesting protective effects of high parity on the risk of AD/D for both fathers and mothers. It implies that parity, as one of the life course contexts, may link to the risk of dementia in later life. More future work is needed to explore potential mechanisms.

